# Nanoscale interfacial gradients formed by the reactive uptake of OH radicals onto viscous aerosol surfaces†
†Electronic supplementary information (ESI) available. See DOI: 10.1039/c5sc02326b


**DOI:** 10.1039/c5sc02326b

**Published:** 2015-09-08

**Authors:** James F. Davies, Kevin R. Wilson

**Affiliations:** a Chemical Sciences Division , Lawrence Berkeley National Laboratory , 1 Cyclotron Road , Berkeley , CA 94720 , USA . Email: KRWilson@lbl.gov

## Abstract

A key but poorly understood chemical process is how gas phase uptake is governed by the relative mobility of molecules at an interface of an atmospheric aerosol. Citric acid (CA), a model system for oxygenated organic aerosol, is used to examine how changes in viscosity, due to changing water content, govern the reactive uptake of gas phase hydroxyl radicals (OH). By comparing the reaction kinetics measured when probing the outer aerosol surface layers with measurements of the bulk particle composition, the effective OH reaction probability is observed to be a complex and non-linear function of the relative humidity (RH). At RH < 50%, the reactive decay of CA is controlled by the viscosity of the particle, where the depletion of CA and the formation of reaction products occurs over a narrow region near the aerosol interface, on the order of 8 nm at 20% RH. At RH = 50% the reaction zone increases to the particle dimensions (*i.e.* ∼50 nm) and at RH > 50%, the aerosol becomes aqueous and well-mixed on the timescale of the heterogeneous reaction. These results imply that in the atmosphere, the formation and dissipation of interfacial chemical gradients could be significant in viscous and semisolid aerosol and play important roles altering gas-particle partitioning and aging mechanisms (*i.e.* bulk *vs.* interface).

## Abstract 2

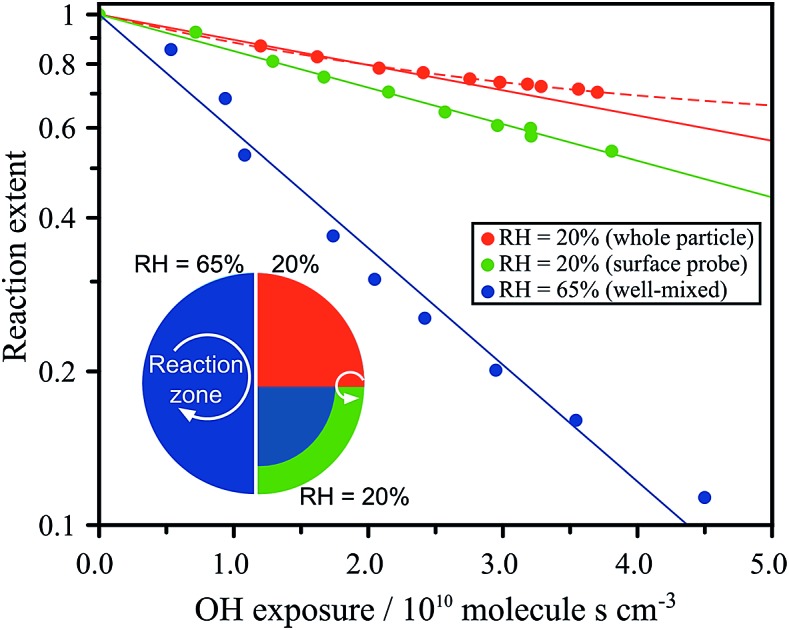
The reaction of hydroxyl radicals with viscous oxygenated organic aerosol forms nanometer-sized interfacial gradients.

## Introduction

**Fig. 1:**
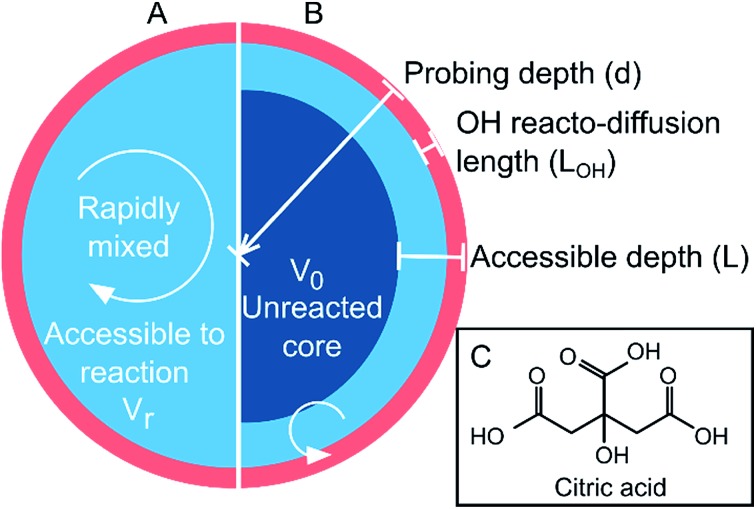
Schematic of (A) a well-mixed droplet; and (B) a droplet with a limited accessible reaction volume defined by depth, *L*. (C) Molecular structure of citric acid.

The chemistry of atmospheric aerosols plays a deterministic role in their physical properties and dynamic behaviour. Chemical changes transform hygroscopicity, toxicity, reactivity, phase state, morphology and aerosol optical properties.^1^–^5^ Thus, understanding the interplay between the physical properties of aerosol and the chemical dynamics associated with oxidative aging is vital in order to quantify their radiative effects and atmospheric lifetimes.^6^–^8^ For simplicity, aerosol particles are treated in climate models as well-mixed and homogeneous,^9^ as illustrated schematically in Fig. 1A. Increasing work has shown that this approximation may be incorrect due to thermodynamic phase separation or kinetic limitations that prevent diffusive mixing over atmospherically relevant timescales.^10^–^18^ The formation of ultra-viscous, semi-solid and glassy states have been reported in atmospheric proxies, such as sucrose and levoglucosan,^18^–^20^ as well as in secondary organic matter (SOM).^21^–^23^ The resulting diffusion limitations may have significant ramifications for reactions that occur at the aerosol surface following collisions with gas-phase molecules (*e.g.* oxidants). The rate of diffusion of a species within the particle (*i.e.* both the oxidant and the organic) can govern the overall rate of chemical processing and gas-particle partitioning through the formation of interfacial chemical gradients.^24^–^29^ This is illustrated in Fig. 1B as an aerosol with an effective core–shell morphology, where the reaction or uptake is confined only to the outer region.

In this work, we measure the heterogeneous oxidation of citric acid (CA) aerosol by hydroxyl radicals (OH). CA, whose molecular structure is shown in Fig. 1C, is used as a model for highly oxygenated material in the atmosphere. The water activity and density of CA solutions have been explored experimentally and are treated explicitly in Extended Aerosol Inorganic Model (E-AIM) as a correction to the UNIFAC approach (Fig. S3A†).^30^–^32^ Furthermore, the viscosity of a CA solution is known to increase as a function of diminishing water concentration, allowing the corresponding diffusion coefficients to be estimated using the Stokes–Einstein relationship (Fig. S3B†),^33^ as described in the ESI.† Citric acid aerosol therefore replicates many of the observed dynamics of SOM and provides an excellent model system in order to explore the role of viscosity on chemical kinetics. The heterogeneous reaction of CA aerosol with OH is measured using high-resolution mass spectrometry as functions of particle size, relative humidity (RH) and probing depth, revealing a complex relationship between the aerosol liquid water content and OH reaction kinetics. A framework based on an accessible reaction volume is used to explain the results, and reveals that the chemistry at RH < 50% is controlled by the formation of nanometer-sized interfacial reaction zones whose dimensions scale with particle viscosity. Analysis of the reaction products indicate that further chemical processing is also influenced by the viscosity of the system.

## Results

**Fig. 2:**
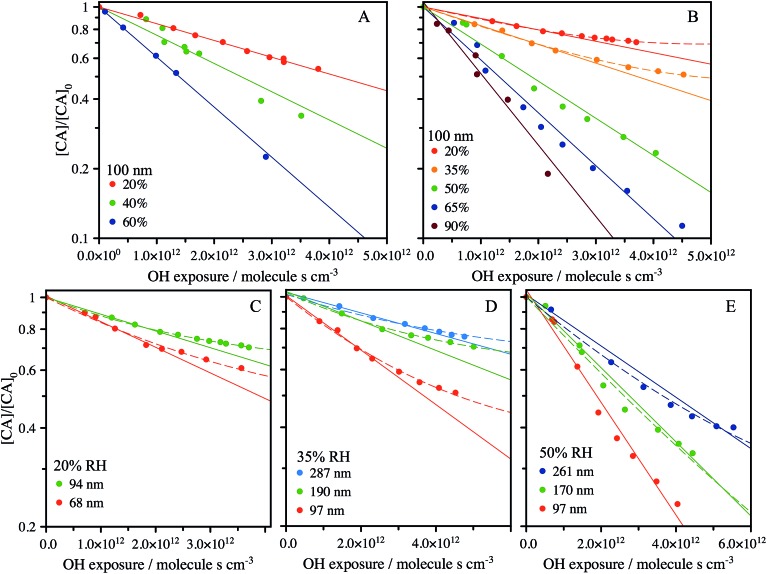
Reactive decay of 100 nm size-selected CA aerosol (inferred from the intensity of the peak at *m*/*z* 191.02) *vs.* RH (20% to 90%) using: (A) the DART heater to probe the surface of the aerosol during ionisation; and (B) an oven to fully vaporise the aerosol prior to ionisation. Kinetic decay plots for fully vaporised size-selected CA aerosol at 20% RH (C), 35% RH (D) and 50% RH (E). A pure exponential decay function is shown by the solid lines, with low RH data fit only at early exposure. A fit to the accessible volume model is shown by the dashed lines, with best fit *k* and *L* tabulated in Table 1.

The heterogeneous reaction of OH with CA aerosol is measured using Direct Analysis in Real Time (DART) ionization mass spectrometry coupled to a flow-tube reactor, as detailed in the Methods section. The change in signal corresponding to CA composition in the aerosol is used to quantify the uptake kinetics and is measured using the *m*/*z* = 191.02 (M – H^–^) peak in the mass spectrum. The decay of this peak is monitored as a function of OH exposure ([OH] × time) with varying RH and particle size. Fig. 2A shows the decay of the CA signal in 100 nm diameter particles using an experimental configuration that vaporizes only the outermost layers of the aerosol (Fig. 1), as described in Chan *et al.*^34^ and in the Methods section below. The probing depth is determined from the measured changes in particle size as the aerosol exits the DART ionization region. As shown in Fig. S2,† the radial probing depth is ∼14 ± 5 nm. Fig. 2A shows that the decay of CA is exponential (linear on a log scale) under all RH conditions when probing only the interface, with a diminishing rate as the RH decreases.

An alternative experimental configuration is used to compare how the observed heterogeneous kinetics change when probing the composition of the entire aerosol, instead of only the outer layers as described above. To measure the composition of the entire particle, the aerosol is passed through a separate oven prior to ionization and detection by the mass spectrometer. To determine the optimum temperature, the oven is increased in 10 °C increments up to 180 °C, where the MS signal corresponding to CA is maximized, and SMPS measurements reveal full vaporization of the aerosol. Under this configuration, as shown in Fig. 2B for RH = 50–90%, the signal decays exponentially and approaches zero with increasing OH exposure, as was observed when probing only the outmost layers of the particle in Fig. 2A. At lower RH (<50%), CA is consumed more slowly and its decay kinetics become non-exponential at exposures of ∼2.5 × 10^12^ molec. s^–1^ cm^–3^. Using the fully vaporized method, the influence of particle size on the oxidation kinetics is measured at RH = 20%, 35% and 50% and is shown in Fig. 2C–E. At the lower RH's, again non-exponential kinetics are observed, with a clear size-dependence in the relative CA signal where the decay deviates from a purely exponential form.

From a comparison of Fig. 2A and B, it is clear that there is always a larger fraction of CA detected, at a given OH exposure, when the composition of the entire volume of the particle is measured. For example, at RH = 20% and [OH] × *t* = 3 × 10^12^ molec. s^–1^ cm^–3^, [CA]/[CA]_0_ = 0.75 for the fully vaporized particle compared to [CA]/[CA]_0_ = 0.59 when only probing the outer ∼14 nm of the particle. The influence of increased mixing due to thermal effects on the partially vaporized signal is not considered here, although may influence the location within the particle from which the signal primarily arises. However, the difference between the partial and fully vaporized data suggests that there is enhanced depletion of CA at the aerosol interface relative to the bulk and is therefore evidence for the formation of chemical gradients in the particle at RH = 20%. Fig. 2C–E show measurements of the decay kinetics of CA as a function of RH and particle size. At RH = 20% and 35% (Fig. 2C and D), the decay kinetics are observed to be non-exponential for all diameters measured. The location on the *y*-axis ([CA]/[CA]_0_) where the decay kinetics deviate from exponential behaviour depends upon particle size, with larger sizes showing a trend towards more unreacted CA. Due to the scaling of surface area and volume with the particle diameter (proportional to *D*^2^ and *D*^3^, respectively), this size dependence is further evidence for an interfacial (*i.e.* surface) component, which controls the observed kinetics. At RH = 50%, the particle size-resolved kinetics all appear exponential within the error of the measurement.

As illustrated by the complete data set shown in Fig. 2, there are three distinct factors which are observed to influence the rate and functional form (exponential *vs.* non-exponential) of the decay kinetics of CA with OH: the particle diameter, RH and the probing method (bulk *vs.* interface). On a molecular level, one might expect that the reactivity of CA with OH to be governed by collision energetics and independent of particle size and RH. However, previous studies have observed similar non-exponential kinetics in droplets and films and qualitatively attribute this effect to diffusive limitations caused by high viscosity.^26^,^35^,^36^ The results shown in Fig. 2 suggest that water plays a considerable role in controlling the uptake kinetics both through its influence on the concentration of CA in the aerosol and by controlling the accessible reactive volume through changes in aerosol viscosity. Below we develop a self-consistent quantitative model of the full data set to better elucidate the role water plays in controlling the heterogeneous reaction of CA with OH.

## Discussion

For the specific case of a well-mixed aerosol particle undergoing heterogeneous oxidation, the reactive decay kinetics are described by a simple exponential function.^37^ In the more general case where the reaction volume is not equal to the particle volume, only the accessible volume will decay exponentially, with a portion or core of the particle remaining unreacted. The reactive decay of CA for this general case is:1
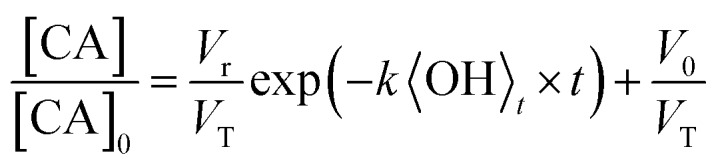
where the accessible reaction volume within the probing region, *V*_r_, and unreacted volume within the probing region, *V*_0_, are expressed as a fraction of the total aerosol volume probed, *V*_T_, with the reaction rate constant, *k*. For the CA reaction with OH described in Results, these volumes are defined by the particle diameter, *D*, a radial reaction depth over which the particle is well-mixed, *L*, and a radial probing depth, *d*, to give:2
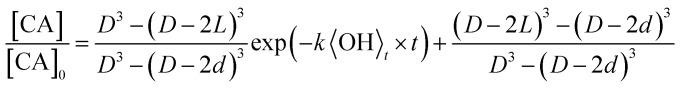
for the case where *L < d*. When *L* > *d*, these parameters are set equal and a standard exponential is obtained. *L* is distinct from the OH reacto-diffusion length, *L*_OH_, which is the distance a gas phase species travels in the particle before reaction. OH is highly reactive and thus has a length < 1 nm. Instead, the magnitude of *L* is governed by diffusion of the organic component rather than OH, and describes the radial depth from which organic molecules in the particle are able to reach the OH surface reaction zone.^27^ When *L* = *D*/2, the particle is well-mixed and a simple exponential function is recovered.

The accessible volume formulation in eqn (2) is used to analyze the reactive decay of CA shown in Fig. 2. Initially, *L* is set equal to the radius of the particle, allowing only *k* to vary. As expected, for the fully and partially vaporized data sets at RH > 50%, the best fit rate constants (at a specific RH) are equal within fitting error. At RH < 50%, the data from the fully vaporised method exhibits a clear deviation from exponential behaviour, as described in Results, while the partially vaporised data retains its exponential form. For these cases, the model values of *k* and *L* are allowed to vary independently to achieve a best fit to the dataset, with *L* constrained to values between zero and the particle radius. Since *L* and *k* are decoupled variables in the model, a unique fit to the data at each size and RH can be obtained. As an internal check of model self-consistency, the values of *L* and *k* obtained from fitting the fully vaporized measurements were used to predict the decay expected for the partially vaporized experiments using a measured probing depth of 14 nm. This comparison exhibits good agreement between model and measurement in the low RH cases, as shown in Fig. 2A, thus validating the self-consistency of *L* and *k* across the entire data set. The best fit model parameters of these data are shown in Table 1.

**Table 1:** Physical properties of CA and kinetic data associated with the OH-initiated oxidation chemistry. Rows in italics indicate data from partially vaporised particles with *d* = 14 nm

RH	*D*/nm	*ρ*_aq_/g cm^–3^	*m*_f_	*k* × 10^13^/cm^3^ s^–1^ molec.^–1^	*L*/nm	*γ*_eff_
21.4	68	1.57	0.89	3.27 ± 0.21	9	0.055
21.3	94	1.57	0.89	3.39 ± 0.13	7	0.052
*20.0*	*100*	*1.57*	*0.89*	*3.31 ± 0.06*	*8*	*0.052*
34.8	97	1.52	0.83	3.21 ± 0.10	14	0.077
35.6	190	1.52	0.83	2.77 ± 0.23	16	0.086
38.8	287	1.51	0.82	2.14 ± 0.18	22	0.092
*40.0*	*100*	*1.50*	*0.81*	*3.09 ± 0.08*	*14*	*0.088*
48.5	97	1.47	0.78	3.95 ± 0.08	44^*a*^	0.135
48.0	170	1.47	0.78	2.87 ± 0.23	54	0.164
48.0	261	1.47	0.78	2.67 ± 0.17	40	0.164
*60.0*	*100*	*1.42*	*0.72*	*4.98 ± 0.05*	*50* ^*a*^	*0.157*
64.5	97	1.40	0.71	5.27 ± 0.28	44^*a*^	0.156
80.0	100	1.31	0.57	6.36 ± 0.06	50^*a*^	0.146
90.0	96	1.22	0.44	7.10 ± 0.26	43^*a*^	0.112

^*a*^Lower limit of estimate (particle well mixed).

**Fig. 3:**
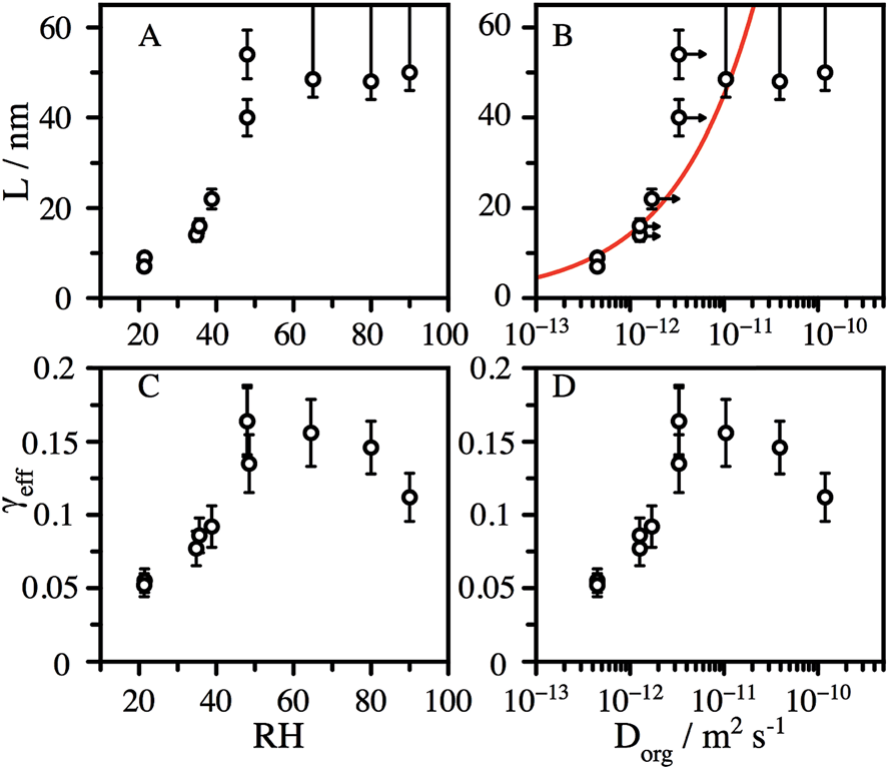
Accessible reaction depth, *L*, inferred from the reactive decay kinetics of CA with OH as a function of: (A) the RH, and (B) the diffusion constant estimated using the Stokes–Einstein relationship. Horizontal arrows show the change in *D*_org_ when corrected for water uptake during the reaction. The red line shows the square-root dependence of *L* on *D*_org_. Effective uptake coefficient, *γ*_eff_, as a function of: (C) the RH, and (D) the diffusion constant.

We observe that *L* is broadly independent of the particle diameter, but varies with the RH, increasing from ∼8 nm at 20% RH to 50 nm at 50% RH (Fig. 3A). The values of *L* at low RH are strongly correlated with the organic diffusion coefficient, *D*_org_, (Fig. 3B), estimated from the particle composition and viscosity (as described in the ESI†). In addition to the decay of CA, we observe particle growth over the course of reaction for RH > 50% (Fig. S4†), suggesting that the formation of reaction products further increases water uptake by the aerosol. Water acts as a plasticizer in hygroscopic viscous aerosol, leading to a more fluid reaction zone and faster diffusion, which is consistent with a significant increase in *L* at elevated RH. We account for this by assuming that the change in mass fraction of organic in the aerosol, due to water uptake, changes the viscosity in the same way as pure CA. This correction shifts the diffusion coefficients to slightly larger values, shown as arrows in Fig. 3B. Although, the formation of smaller molecules by reaction with OH reaction may also reduce the aerosol viscosity and increase the diffusive mobility of CA, for simplicity this factor is not explicitly considered in the model.

The heterogeneous rate constant determined from fits to the decay curves varies significantly with both particle size and RH (Fig. S5†). As described in the ESI,† *k* is used to compute an effective uptake coefficient, *γ*_eff_, which is the fraction of collisions of OH with CA molecules at the particle surface that result in a reaction.^37^,^38^ Taking into the account the concentration using the mass fraction, *m*_f_, and the density, *ρ*_aq_, of CA, the rate constant is related to *γ*_eff_*via*:3
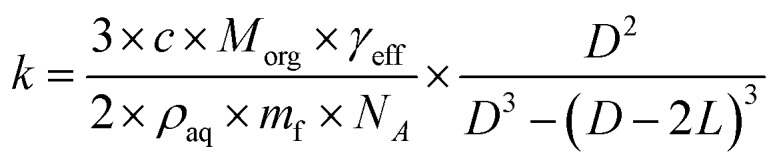
where *N*_A_ is Avogadro's number, *c* is the mean velocity of OH and *M*_org_ is the molecular mass of the organic component. This equation is used to compute *γ*_eff_ from the measured *k*, which are shown in Fig. 3C and D as a function of RH and the estimated CA diffusion coefficient, *D*_org_. Two distinct regions are observed. At high RH, *γ*_eff_ increases with decreasing RH, reaching a maximum value of 0.15 at 50% RH, and subsequently decreases to 0.05 at 20% RH. The high RH dependence of *γ*_eff_ is explained by the simple dilution of CA at the surface by water. This leads to OH collisions with surface water molecules that do not result in reaction with CA, consistent with the observations reported by Slade and Knopf for OH uptake onto methyl-nitrocatechol.^25^ The low RH dependence of *γ*_eff_ is explained by the increased viscosity in the particle phase. Previous studies observed that the uptake coefficient of OH onto levoglucosan aerosol was correlated with the viscosity of the sample and attributed these changes to diffusive limitations on the mobility of OH in solution.^25^

**Fig. 4:**
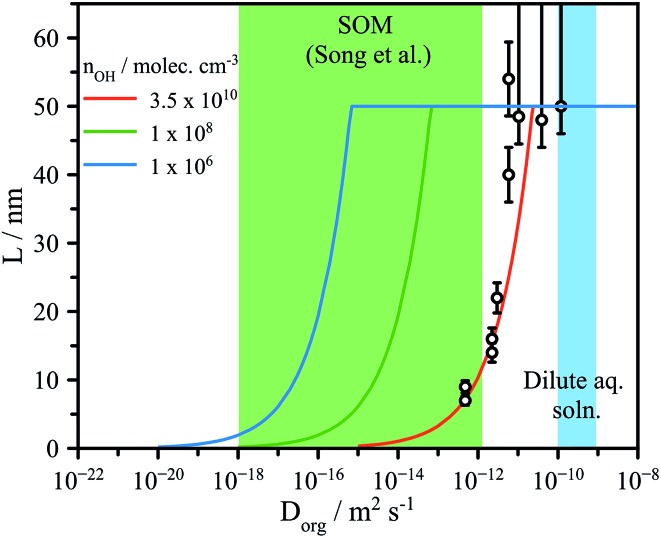
Experimentally determined reaction depth (*L*) compared to model predictions (eqn (4)) using the formulation of Houle *et al.*^27^ for various [OH] ranging from flow tube to atmospheric concentrations (10^6^ molec. cm^–3^). Green shaded region indicates the diffusion coefficients expected for dilute aqueous solutions and for secondary organic matter (SOM), as reported by Song *et al.*^41^

Establishing the origin of the competing timescales that couples surface reactions with diffusion limitations is an ongoing challenge. For a model alkane system, Houle *et al.*^27^ proposed a kinetic clock in which the reaction depth, *L*, is governed by *D*_org_, *γ*_eff_ and the time between reactive OH reactive collisions with the particle,4
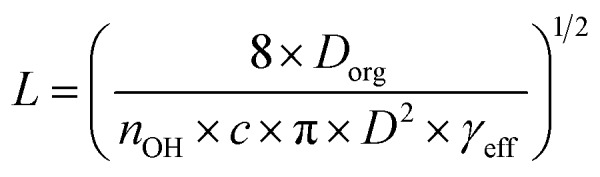
where *n*_OH_ is the average OH concentration. This formulation predicts a diameter dependence of *L*, arising from the dependence of diffusion timescale on the collision frequency. We do not see a clear size dependence, which may indicate another factor regulates the timescale, independent of collision frequency. It may also be the case that the size dependence is obscured by other factors unique to the CA system that are not considered in the simple model formulation. Since the CA aerosol is hygroscopic, *L* could also depend upon the water diffusion kinetics in addition to the OH reactive collision frequency. It is also possible that as the reaction progresses, the viscosity in the outer layer evolves differently than the core of the aerosol comprised mainly of CA. These are more complex feedbacks between reaction, water uptake, and viscosity than were originally considered for the non-hygroscopic alkane system described by Houle *et al.* Nevertheless, for a particle diameter of 100 nm, the estimated *L* predicted by eqn (4) is within ±20% of the experimentally derived values for RH < 50%, as shown in Fig. 4.

In addition to the decay of CA, high resolution mass spectrometry is used to measure how the reaction products and average elemental composition of the aerosol evolves with reaction and RH. Average changes in elemental composition are represented as slopes in a van Krevelen diagram, which reveals how the oxygen-to-carbon (O/C) and hydrogen-to-carbon (H/C) ratios evolve with reaction. The slope in the van Krevelen diagram reflects average differences in oxidation pathways.^39^ A slope of zero suggests alcohols are formed in the reaction in contrast with a slope of –2, which reflects the formation of ketone functional groups. Typically, aerosol undergoing OH-initiated oxidation exhibit a slope around –1, indicating a mixture of ketone and alcohol functional groups (also carboxylic acids) are produced by the reaction.^24^,^39^

**Fig. 5:**
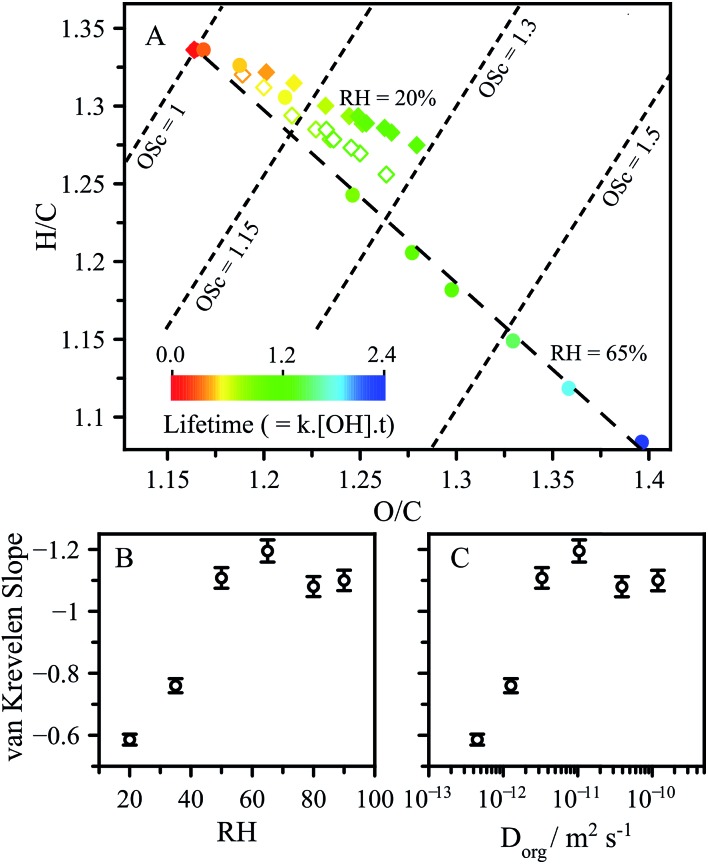
(A) van Krevelen diagram showing oxidation at RH = 65% (solid circles), RH = 20% (solid diamonds). RH = 20% with the contribution of the functionalised alcohol removed (open diamonds), as described in the text. Slope of the product data in van Krevelen space as a function of: (B) and (C) diffusion coefficient.

As shown in Fig. 5A, the CA reaction proceeds along a slope of around –1 at RH > 50%, as is expected for typical free radical oxidation pathways (*i.e.* formation of ketone and alcohol reaction products). In contrast, the slope at 20% RH is –0.6, representing a significantly different product distribution than observed under higher RH conditions. The dependence of the slope on RH and diffusion coefficient are shown in Fig. 5B and C.

These slopes exhibit the same distinctive dependence observed in Fig. 3 for *L* and *γ*_eff_ as a function of RH and *D*_org_. Further molecular analysis of the products and their kinetic evolution at 20% RH (presented in the ESI.†) reveals a continuous build-up of higher molecular weight reaction products, corresponding to a CA molecule with new oxygenated (alcohol or ketone) functional groups. This is in contrast to the 65% RH case, where these same higher molecular weight products reach maxima and then decrease as the reaction proceeds. This observation suggests that for these functionalised products the accessible reaction depth is smaller than for CA, which may arise due to slower diffusion of the more oxygenated products. As a result, these products become less available for reaction in a viscous solution as they are less able to reach the surface on the reaction timescale. This is illustrated by removing the elemental contribution of the major functionalised product corresponding to the alcohol (*m*/*z* 207.01) from the van Krevelen analysis. Removal of the contribution from this product produces a slope of –0.87, which is much closer to the 65% RH case of around –1.1. This is in contrast to the high RH case where the removal of the same alcohol changes the slope in van Krevelen space by <5%. The differences in van Krevelen slope which arise as a function of RH are related to the same diffusion limitations which lead to chemical gradients, and represents another consequence of viscosity on aerosol chemistry. One further metric used to report changes in chemical composition is the average carbon oxidation state (OS_c_), obtained from the relationship OS_c_ = 2 × O/C – H/C.^24^,^40^ At a given kinetic lifetime, OS_c_ remains unchanged as a function of RH, as seen from the OS_c_ contours presented in Fig. 5A and exemplified in Fig. S8† showing the distributions of OS_c_ for individual products in the aerosol.

The analysis of the reaction products presented here suggests that the underlying reaction mechanism of CA with OH is not strongly influenced by the availability of water, but rather the elemental composition of the aerosol is primarily controlled by the ability of first generation reaction products to diffuse to the surface and undergo further reaction with OH. Therefore, the relationship between the reactive uptake coefficient and RH seems to arise from diffusive limitations rather than any changes in reaction mechanism.

## Conclusions

We have demonstrated the influence of aerosol water content and viscosity on gas phase uptake using OH-initiated heterogeneous chemical processing to probe a model oxygenated aerosol. We have explored two major consequences of viscosity on chemical kinetics in aerosol. Firstly, the dependence of reactive uptake kinetics on the particle phase is clear from the low reported *γ*_eff_ at RH < 50%, leading to consequences in the initial OH processing and the evolution of the products. Secondly, we have identified the formation of chemical gradients brought about by reaction at the aerosol particle surface and slow bulk phase mixing due to diffusive limitations. The precise role of viscosity in regulating gas-particle interactions on the molecular scale remains an open and unanswered question.

The formation of interfacial gradients in viscous and semisolid aerosol under atmospheric OH concentrations may be evaluated by eqn (4). Despite the lack of a strong observed diameter dependence of *L* in Fig. 4 (possible reasons for which are discussed in detail above), eqn (4) does capture the overall changes in reaction depth *vs. D*_org_ for the experimental conditions used here (*e.g.* [OH], *γ*_eff,_*etc.*). The viscosities observed in secondary organic material representative of the ambient aerosol are up to 8 orders of magnitude larger than those explored here at room temperature, and likely significantly greater at the low temperatures commonly encountered in the troposphere.^15^,^21^,^41^,^42^ Accounting for the lower atmospheric OH concentrations, which are on the order of 10^6^ molec. cm^–3^ (*vs.* 10^10^–10^11^ in the flow tube), eqn (4) suggests that it is highly likely that as organic aerosol in the atmosphere is transformed by OH, significant interfacial gradients will form, as shown in Fig. 4. We predict that for a 100 nm diameter aerosol, reaction with OH will form interfacial reaction gradients of 1–25 nm in semisolid aerosol with values of *D*_org_ between 10^–18^ and 10^–16^ m^2^ s^–1^. Such chemical gradients will in turn play significant roles in controlling further water uptake and aerosol optical properties (core–shell) in the atmosphere. Further work is clearly needed using a range of reactive and non-reactive chemistries and techniques to directly observe the formation and dissipation of chemical gradients at aerosol surfaces to fully ascertain the atmospheric implications for the multiphase chemistry of viscous aerosol. Given the increasing prevalence of multiphase reaction-diffusion models,^27^,^43^ the data reported here are key benchmarks against which model predictions can be evaluated.

## Methods

The ˙OH initiated oxidation of CA aerosol is measured using a flow-tube reactor setup, similar to those described in previous publications and shown schematically in Fig. S9.† 24,34,44 A dilute CA solution was atomized under nitrogen using a TSI Aerosol Generator (3076) and 300 sccm of the resulting flow was passed through a Nafion diffusion drier and a TSI Electrostatic Classifier (3080L) for size-selection of the aerosol. The resulting aerosol flow was mixed with nitrogen (800 sccm), oxygen (150 sccm) and hexane/nitrogen (150 sccm of 5 ppm hexane in nitrogen) gas to a total flow of 1.4 L min^–1^. The flow, given sufficient time to reach equilibration at a known RH, measured by an inline Vaisala capacitance probe, is introduced into a water-cooled quartz flow tube at a temperature of 20 °C. Through an additional gas inlet a mixture of ozone, oxygen and nitrogen was introduced at a flow rate of 100 sccm, with total ozone concentrations ranging 0.5–100 ppm. Ozone is produced using a corona discharge ozone generator and measured using a UV absorption ozone monitor (2B Technology 106-L). The total aerosol concentration was typically in the region of 100–500 μg m^–3^ in a flow of 1.5 L min^–1^, with the geometrical standard deviation in the size-selected diameter of approximately 1%. The flow tube is illuminated by UV light from multiple Hg lamps at 254 nm spanning the length of the flow tube, inducing the formation of OH radicals *via* the photolysis of O_3_. The interaction time in the flow tube is estimated to be 60 s based on the total flow and internal volume. The decay of hexane, measured by gas chromatography, is used to determine the average ˙OH exposure using a relative rates approach described by Smith *et al.*^37^ and a rate constant of 5.2 × 10^–12^ cm^3^ s^–1^ molec.^–1^.^45^ The flow exiting the reactor is analysed by a second TSI Electrostatic Classifier coupled with a butanol TSI Condensation Particle Counter (3772), and a Q Exactive Orbitrap Mass Spectrometer (Thermo Scientific) for exact mass analysis of the aerosol constituents. The ionisation prior to MS analysis is done using an Ionsense Direct Analysis in Real Time (DART SVP) ion source, supplied with helium, used at room temperature in conjunction with an oven to fully vaporise the aerosol, or at elevated DART gas temperatures to desorb material from the particle surface, leading to partially vaporized particles. In both cases the ionisation took place in a glass enclosed volume, which was found to produce a more stable signal over time in the MS than for ionisation in the open laboratory, as has been used in previous studies.^24^,^34^,^44^ The DART ionisation method generates mainly molecular ions, which is one of the major benefits of using such an approach for multi-component analysis of organic aerosol.^34^ The orbitrap is operated in negative ion mode with a mass resolution of 70 000 over the *m*/*z* range 50–500 and spectra were collected at a rate of approximately 1 Hz. All spectra are recorded and averaged over a sampling time of three to five minutes. The method adopted here allows unambiguous molecular identification of the reaction products through an exact mass analysis, and quantification of their relative abundance under the assumption of a constant ionisation efficiency.

## Supplementary Material

Supplementary information
