# Supplementary material for: Nanoscale interfacial gradients formed by the reactive uptake of OH radicals onto viscous aerosol surfaces
Source: Chem Sci. 2015 Sep 8;6(12):7020–7. doi: 10.1039/c5sc02326b (PMC5947524; doi:10.1039/c5sc02326b)
Supplement: Supplementary file 1 [file SC-006-C5SC02326B-s001.pdf]

Electronic Supplementary Material (ESI) for Chemical Science.
This journal is © The Royal Society of Chemistry 2015

Supplementary Information

Nanoscale Chemical Gradients Formed in the Reactive Uptake of OH Radicals by Viscous Organic Aerosol

James F. Davies and Kevin R. Wilson
Chemical Sciences Division, Lawrence Berkeley National Laboratory, 1 Cyclotron Road, Berkeley, CA 94720

Mass Spectral Analysis of CA Aerosol Pre- and Post-Oxidation

The mass spectrum recorded for citric acid aerosol prior to oxidation by OH is shown in Figure S1A. Using helium as the DART source supply gas in the ionisation step following complete aerosol vaporisation at 180°C, the principle ion formed is the single negatively-charged molecular ion, resulting from loss of a proton, at m/z 191.02 ($C_6H_7O_7^-$). There is minimal fragmentation, with a peak at m/z 111.01 ($C_5H_3O_3^-$) at intensity < 2% of the molecular ion peak identified by MS/MS as the only ion-fragment observed. Following oxidation of CA at an OH exposure of 1.0×10^{12} molecule s cm^{-3}, the mass spectrum reveals additional peaks, as shown in Figure S1B. The CA peak at 191.02 remains the most dominant, with the additional peaks developing primarily at lower m/z.

Concentration and Viscosity of CA Aerosol

The particle composition at a given water activity, equivalent to fractional RH when the aerosol is at equilibrium, is estimated using thermodynamic models. Using the modified UNIFAC parameters of Peng et al. (1, 2), implemented through the Extended Aerosol Inorganic Model (E-AIM) framework (3), the concentration and density of a CA aerosol droplet is computed and shown in Figure S3.

The viscosity of CA is estimated by fitting the functional form reported by Chenlo et al. (4) to the data reported by Laguerie et al. (5), shown in Figure S3B as a function of RH. Over the range of RH explored in this study, the viscosity increases by around three orders of magnitude, reaching values of around 2 Pa s at RH = 20% RH, 2000× that of pure water. The corresponding diffusion coefficients were estimated using the Stokes-Einstein relationship with an estimated molecular radius of CA of 0.36 nm.

The reactive uptake kinetics for a well-mixed single component aerosol

While this derivation has been presented elsewhere, it is presented here in the interests of fully clarifying subsequent derivations of more complex systems. Starting with the definition of the reactive uptake coefficient, $\gamma_{eff,}$ for OH onto an organic particle – the fraction of OH collisions with organic molecules which result in reaction – we can write:

$$\frac{d[org]}{dt} = -\gamma_{eff}.f.J_{coll}.C_p.A \qquad \text{(S1)}$$

where [org] is the number concentration of organic molecules in the aerosol, f is the fraction of organic molecules remaining (= $[org]/[org]_0$), J_{coll} is the molecular flux of

OH (= c[OH]/4); where c is the molecular speed of OH), A is the surface area of a particle and C_p is the number concentration of particles in the aerosol.

A second order rate equation may be written for the reaction between OH and organic:

$$\frac{d[org]}{dt} = -k[org][OH] \qquad \text{(S2)}$$

which has an exponential solution describing the decay of the organic, given by:

$$\frac{[org]}{[org]_0} = exp\left(-k\int_0^t [\cdot OH]t\right) \qquad \text{(S3)}$$

where $\int_0^t [\cdot OH]t = \langle \cdot OH \rangle_t.t$ and represents the average ·OH exposure, obtained experimentally by use of a tracer method. The concentrations of reactants here are defined by the total number of molecules per unit area in the aerosol; the quantity determined experimentally. The reaction rate constant, k, represents a heterogeneous process and, thus, is not a fundamental quantity of the system, depending instead on the geometry of the aerosol, in particular the surface area to particle volume ratio. Equating Equations S1 and S2, we obtain:

$$k = \frac{\gamma_{eff}.f.J_{coll}.C_p.A}{[org][OH]} = \frac{\gamma_{eff}.c.A.C_p}{4.[org]_0} \qquad \text{(S4)}$$

Given that the concentration refers to the molecular number concentration of organic molecules in the aerosol, we can write:

$$[org]_0 = \frac{C_p.V.\rho_0.N_A}{M_{org}} \qquad \text{(S5)}$$

where V is the particle volume and M_{org} is the molecular mass of the organic species. This leads to an expression for the heterogeneous rate constant:

$$k = \frac{\gamma_{eff}.c.M_{org}}{4.\rho_0.N_A}.\frac{A}{V} = \frac{3.\gamma_{eff}.c.M_{org}}{2.D.\rho_0.N_A} \qquad \text{(S6)}$$

which is applicable only in the homogeneous, single component case, with particles of diameter, D.

The reactive uptake kinetics for a well-mixed aqueous aerosol

Introducing a second component into the aerosol, in this case water, increases the complexity, and necessitates a number of adaptations to the formulation of kinetic equations. The main difference from the single component case comes in Equation S5, which must be reformulated as:

$$[org]_0 = \frac{C_p.V.\rho_{aq}.m_f.N_A}{M_{org}} \quad \text{(S7)}$$

to take account of the aqueous phase density and the mass fraction of the organic in the particle, denoted by m_f. Thus, k, in the case of an aqueous aerosol, becomes:

$$k = \frac{\gamma_{eff}.c.M_{org}}{4.\rho_{aq}.m_f.N_A}.\frac{A}{V} = \frac{3.\gamma_{eff}.c.M_{org}}{2.D.\rho_{aq}.m_f.N_A} \quad \text{(S8)}$$

We explore the expected influence of dilution on the chemical decay kinetics in Figure S6A for citric acid aerosol under a range of RH conditions. Clearly, for a constant γ_{eff} to apply, the observed rate must increase with increasing RH.

The reactive uptake kinetics for a core-shell aqueous aerosol

When the whole particle is not available for reaction, as in the case where diffusive limitations are present, then some further modifications are made to the formulation of the γ_{eff}. The derivation is almost identical, except the concentration of organic now corresponds to the number of molecules in the accessible volume (indicated by *). Thus, the reaction rate constant derived experimentally must correspond to the reacted portion only, leading to the rate equation:

$$\frac{d[org]^*}{dt} = -k[org]^*[OH] \quad \text{(S9)}$$

Equation S7 then becomes:

$$[org]_0^* = \frac{C_p.V_r.\rho_{aq}.m_f.N_A}{M_{org}} \quad \text{(S10)}$$

where V_r is the volume of the particle which takes part in the reaction, and is defined by the depth into the particle over which full mixing occurs on the timescale of an OH-particle interaction.

The most general formulation of k for aqueous aerosol in any morphology then becomes:

$$k = \frac{3.c.M_{org}.\gamma_{eff}}{2.\rho_{aq}.m_f.N_A}.\frac{A}{V_r} \quad \text{(S11)}$$

The formulation of this equation takes account of all geometry related effects which influence the kinetics. Thus, one can assume for a given chemical system with a constant fundamental rate constant (i.e. the chemistry is the same), the reactive uptake coefficient is constant and the measured reaction rate constant varies according to the geometry and composition. We use Equation S11 to predict how we might expect reactive decay curves to appear for different reaction volumes, defined by an accessible depth L, in Figure S6B. The limited reaction volume results in an increased k as the

surface-to-volume ratio is increased relative to the case where the whole volume reacts.

Products of the oxidation of CA

Due to the limited reaction volume at RH < 50%, the product intensities are normalised against the amount of CA available in the reaction, rather than the total CA signal as measured experimentally. This is a simple rescaling based on the inferred reaction volume given by the depth, *L,* and results in an enhancement in the amount of each product relative to the measured signal. All analysis of the products discussed here consider only the reaction region in both cases in order to compare the chemistry in the absence of the influence of the unreacted core.

To explore the role of water on the chemical pathways available in the OH-initiated oxidation of CA aerosol, the products of the high RH (65%) and low RH (20%) reaction are compared. The majority of the observed products arise from fragmentation pathways leading to a large number of peaks (m/z< 191.02) with carbon numbers less than 6. There are two notable peaks at higher m/z at 205.00 ($C_6H_5O_8^-$) and 207.01 ($C_6H_7O_8^-$). The latter corresponds to the addition of an alcohol group to CA, while the peak at 205 may correspond to the ketone equivalent, or a functionalised epoxide formed by dehydration of the alcohol and further functionalisation. Another notable functionalisation peak, which is only seen at high RH, is m/z 189 and likely corresponds to the epoxide formed directly from the alcohol, with no additional functionality. The kinetic evolution of these products is shown in Figure S7A on a CA lifetime axis to normalise the data based on the extent of reaction.

At high RH, the signals at m/z 205 and 189 rise subsequent to an increase in the signal at m/z 207.01, suggesting they arise from further reaction of the alcohol. At low RH, there is no signal at m/z 189, and the signals at m/z 205 and 207.01 evolve together, suggesting, in this case, that m/z 205 corresponds to the first-generation ketone product. Formation of ketone functionality in this position leads to a structure which can easily undergo decarboxylation, which may explain the lack of signal for the ketone in all conditions, which would be expected to form with a more significant yield when considering standard OH-initiated oxidation pathways (6–8). In the condensed phase, the ratio of alcohol to ketone depends on temperature and viscosity and, thus, a one-to-one ratio is not be expected (9). Fragmentation products arise following β-scission of the alkoxy radical and produce lower molecular weight products. The dominant fragmentation products seen experimentally have a three-carbon backbone, a molecular structure which cannot be formed directly by fragmentation of CA. This suggests that significant fragmentation of the first-generation products occurs, allowing such structures to form. An alternative explanation relies on chemistry occurring beyond the standard pathways available, which is possible given the high degree of functionality present in CA, and the very low pH and high concentration of the solution. A full list of the products observed for CA oxidation, and their most likely structure based on the reaction mechanisms available, is given in Table S1.

At high RH, the functionalised products increase to a maximum before subsequently falling off in intensity, as the fragmentation products grow to dominate the total intensity (Figure S7A). At low RH, the product signals increase together, and no maximum is reached in the intensity of the functionalised products. The simultaneous

formation of products was observed by Chan et al. for solid succinic acid particles and may be rationalised by the decreased mobility of functionalised products due to the presence of additional H-bonding groups, particularly in the case of the alcohol product at m/z 207.01. This leads to a build up of the functionalised products within the particle which are unable to undergo further reaction with OH due to their lower diffusivity in the solution.

1. Peng C, Chan MN, Chan CK (2001) The hygroscopic properties of dicarboxylic and multifunctional acids: measurements and UNIFAC predictions. *Env Sci Technol* 35(22):4495–4501.

2. Peng C, Chow AHL, Chan CK (2000) Study of the Hygroscopic Properties of Selected Pharmaceutical Aerosols Using Single Particle Levitation. *Pharm Res* 17(9):1104–1109.

3. Wexler AS, Clegg SL (2002) Atmospheric aerosol models for systems including the ions H+, NH4+, Na+, SO42−, NO3−, Cl−, Br− and H2O. *J Geophys Res* 107(D14).

4. Chenlo F, Moreira R, Pereira G, Ampudia A, Rosa D (2002) Viscosities of aqueous solutions of sucrose and sodium chloride of interest in osmotic dehydration processes. *J Food Eng* 54(4):347–352.

5. Laguerie C, Aubry M, Couderc J (1976) Some physicochemical data on monohydrate citric acid solutions in water: solubility, density, viscosity, diffusivity, pH of standard solution, and refractive index. *J Chem Eng Data* 21(1):85–78.

6. Tyndall G, Cox R (2001) Atmospheric chemistry of small organic peroxy radicals. *J Geophys Res Atmos* 106:12157–12182.

7. Orlando JJ, Tyndall GS (2012) Laboratory studies of organic peroxy radical chemistry: an overview with emphasis on recent issues of atmospheric significance. *Chem Soc Rev* 41(19):6294–6317.

8. Atkinson R, Arey J (2003) Atmospheric degradation of volatile organic compounds. *Chem Re* 103:4605–4638.

9. Bennett JE, Summers R (1974) Product Studies of Mutual Termination Reactions of Sec-Alkylperoxy Radicals - Evidence for Non-Cyclic Termination. *Can J Chem* 52:1377–1379.

10. Davies JF, Haddrell AE, Reid JP (2012) Time-resolved measurements of the evaporation of volatile components from single aerosol droplets. *Aerosol Sci Technol* 46(6):666–677.

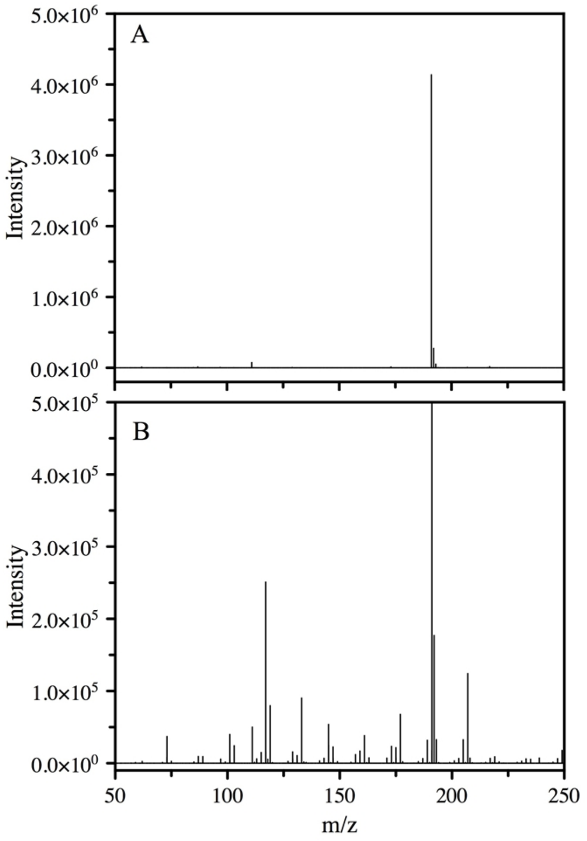

Figure S1: (A) Mass spectrum recorded for 100 nm size-selected citric acid aerosol at a mass loading of ~100 µg m^{-3}, with a clear peak at 191.02 and a smaller peak at 111.01 arising from the molecular ion of CA and an ion fragment. (B) Mass spectrum recorded for the same aerosol exposed to OH at 1.0 × 10^{12} molecule s cm^{-3}, with additional peaks clearly seen at higher and lower m/z.

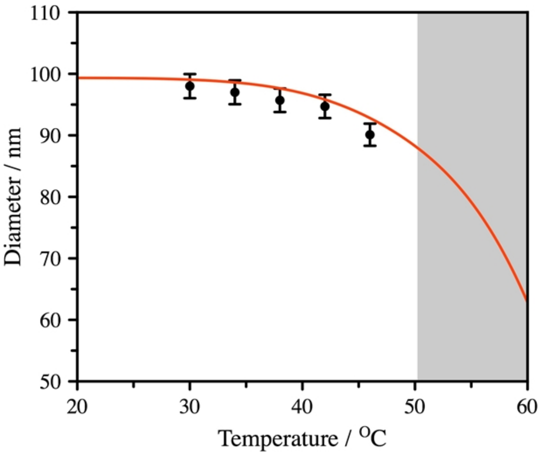

Figure S2: Mean diameter of CA aerosol initially at 100 nm exposed to DART ionisation region temperatures under nitrogen (black points). The red line shows the estimated size change given the change in vapour pressure according to the Clausius-Clapeyron relationship and the mass-flux equations described in Davies et al. (10). The shaded region indicates the temperature in the ionisation region using helium.

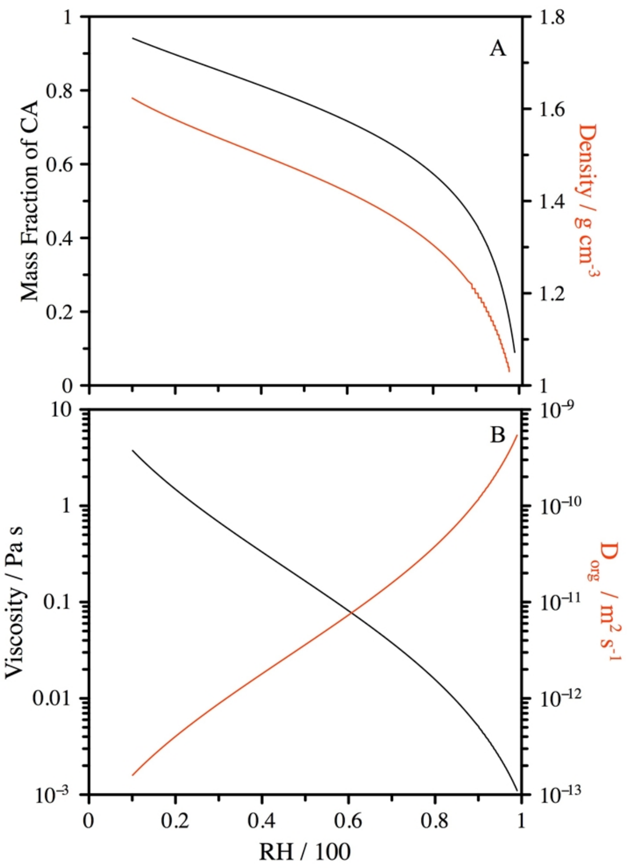

Figure S3: The water activity dependence for a range of properties associated with aqueous citric acid, estimated using a combination of E-AIM, the equations of Chenlo et al. and the Stokes-Einstein relationship, as described in the text.

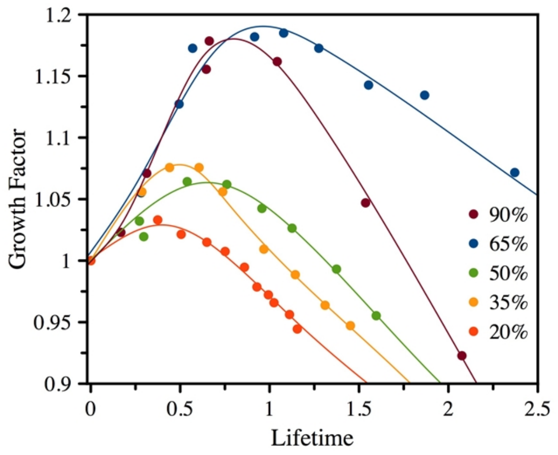

Figure S4: Change in relative diameter of CA aerosol during the OH initiated oxidation reaction under various RH conditions.

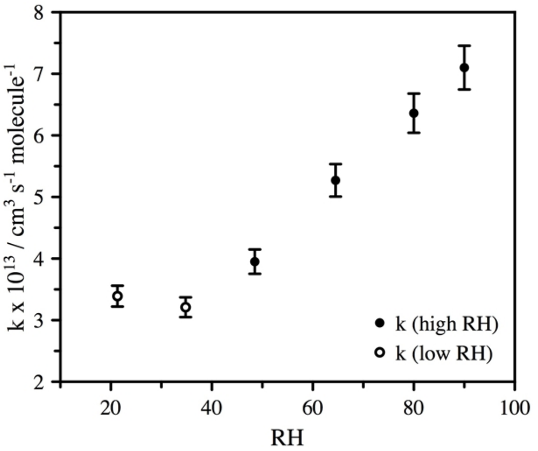

Figure S5: Heterogeneous rate constants for the reaction between 100 nm CA aerosol and OH, determined using the accessible volume framework for RH < 50%, and the inferred accessible depth, *L*.

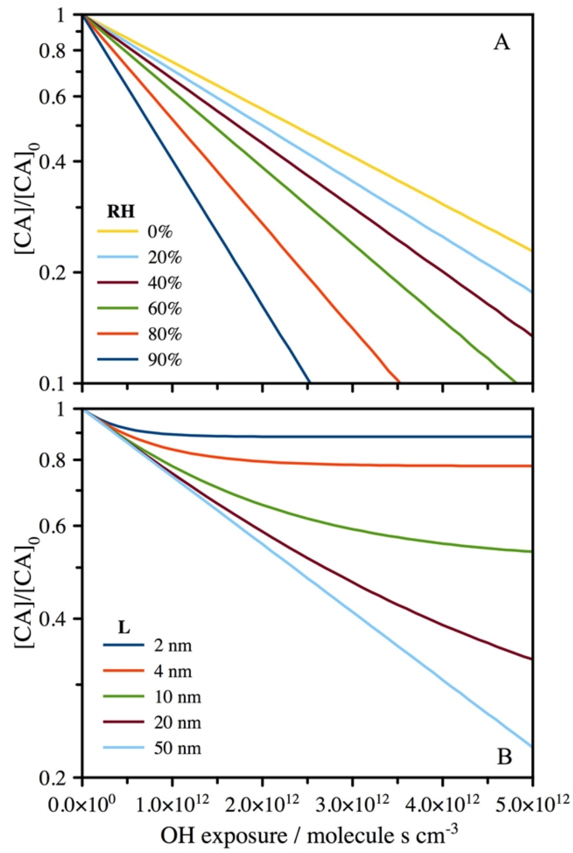

Figure S6: (A) Expected kinetic decay for CA under different RH conditions given an uptake coefficient of 0.15. (B) Expected kinetic decay for pure CA for varying reaction depth, *L*, for the same fixed uptake coefficient.

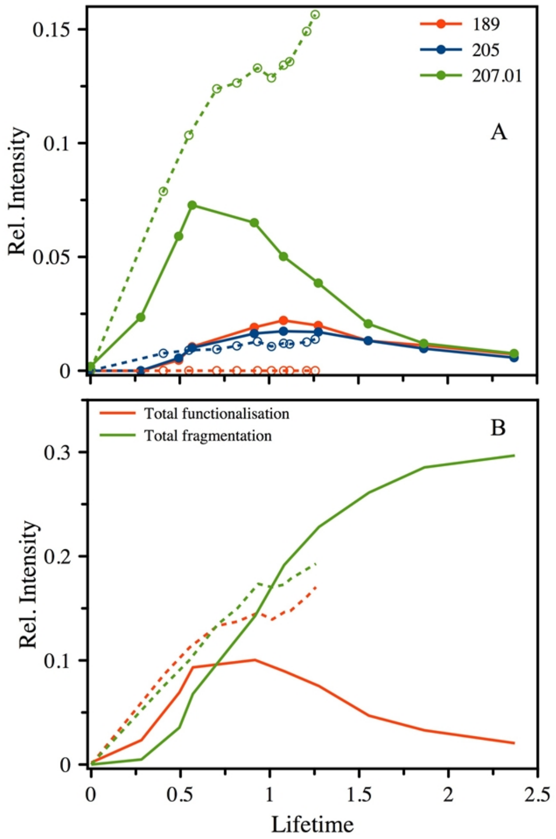

Figure S7: (A) Variation in the intensity of reaction product peaks corresponding to functionalised products at m/z 207.01, 205.00 and 189.00 at 65% RH (solid circles and lines) and 20% RH (open circles, dash lines). (B) Total signal corresponding to functionalised and fragmented products at 65% RH (solid lines) and 20% RH (dash lines).

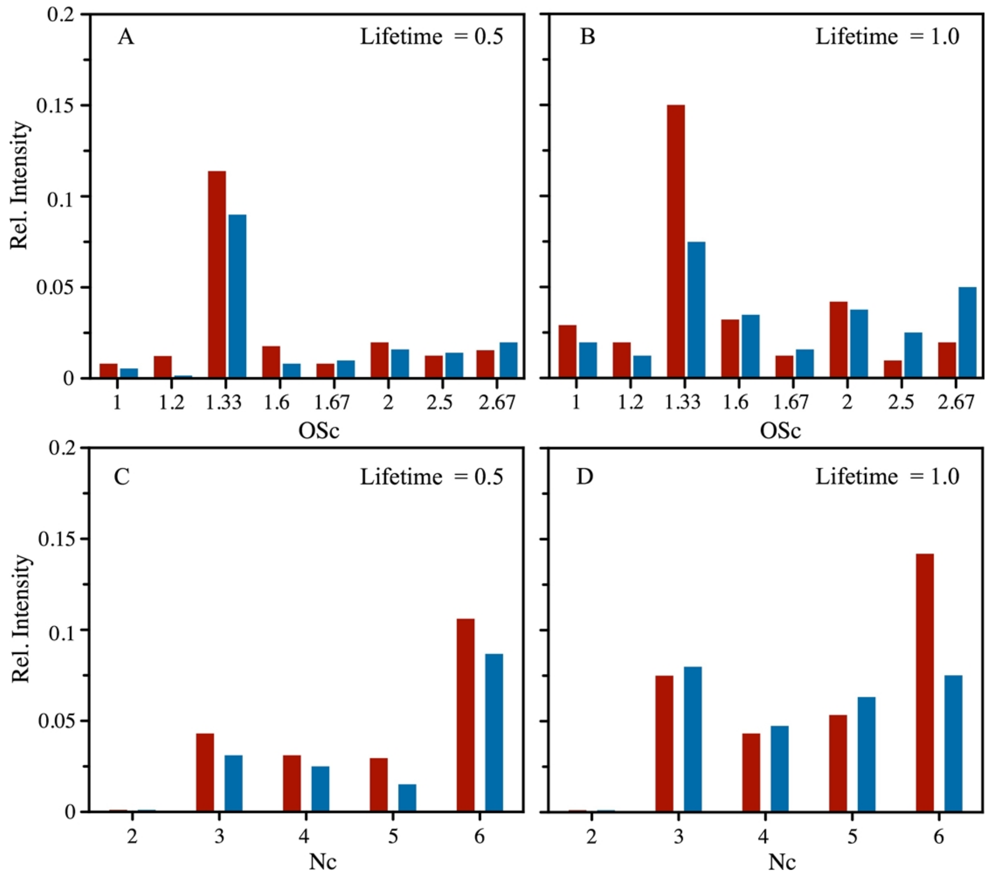

Figure S8: (A) and (B): Molecular oxidation state distributions at a CA lifetime of 0.5 and 1.0 for: 20% RH (red) and 65% RH (blue). (C) and (D): Analogous carbon number distributions.

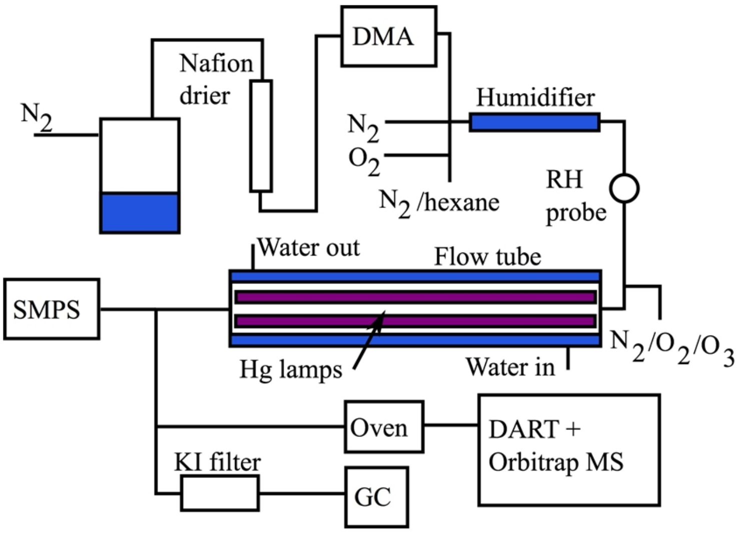

Figure S9: Experimental flow-tube setup.

Table S1: Products of the OH-initiated oxidation of CA.

m/z	-ve ion fragment	Structure	H/C	O/C	m/z	-ve ion fragment	Structure	H/C	O/C
100.99	C_3HO_4	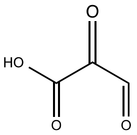	0.67	1.33	161.01	$C_5H_5O_6$	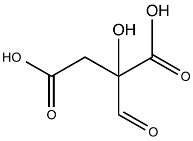	1.20	1.20
103	$C_3H_3O_4$	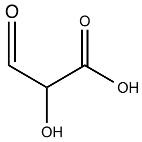	1.33	1.33	177	$C_5H_5O_7$	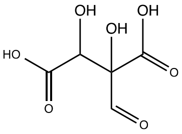	1.20	1.40
116.98	C_3HO_5	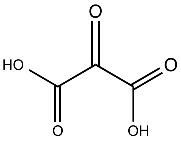	0.67	1.67	189	$C_6H_5O_7$	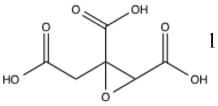	1	1.67
119	$C_3H_3O_5$	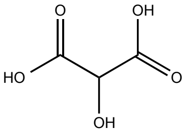	1.33	1.67	**191.02**	**$C_6H_7O_7$**	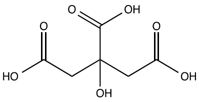	**1.33**	**1.17**
133.01	$C_4H_5O_5$	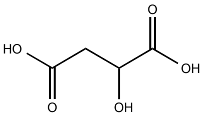	1.50	1.25	205	$C_6H_5O_8$	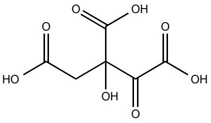	1.00	1.33
144.98	C_4HO_6	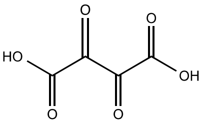	0.50	1.50	" "	" "	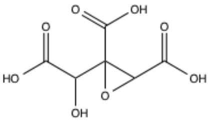	" "	" "
145.01	$C_5H_5O_5$	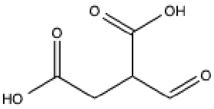	1	1.2	207.01	$C_6H_7O_8$	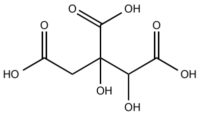	1.33	1.33
146.99	$C_4H_3O_6$	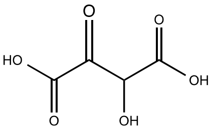	1.00	1.50					
